# e-Transmission of ECGs for expert consultation results in improved triage and treatment of patients with acute ischaemic chest pain by ambulance paramedics

**DOI:** 10.1007/s12471-018-1187-0

**Published:** 2018-10-24

**Authors:** S. S. Anroedh, I. Kardys, K. M. Akkerhuis, M. Biekart, B. van der Hulst, G. J. Deddens, P. Smits, M. Gardien, E. Dubois, F. Zijlstra, E. Boersma

**Affiliations:** 1 Department of Cardiology, Erasmus MC, Rotterdam, The Netherlands; 2 Cardiovascular Research school COEUR, Erasmus MC, Rotterdam, The Netherlands; 3 Ambulance service Rotterdam-Rijnmond region, Barendrecht, The Netherlands; 4 Department of Cardiology, Maasstad Ziekenhuis, Rotterdam, The Netherlands

**Keywords:** Myocardial infarction, NSTEMI, STEMI, ECG, Diagnosis, Primary PCI

## Abstract

**Aims:**

In pre-hospital settings handled by paramedics, identification of patients with myocardial infarction (MI) remains challenging when automated electrocardiogram (ECG) interpretation is inconclusive. We aimed to identify those patients and to get them on the right track to primary percutaneous coronary intervention (PCI).

**Methods and results:**

In the Rotterdam-Rijnmond region, automated ECG devices on all ambulances were supplemented with a modem, enabling transmission of ECGs for online expert interpretation. The diagnostic protocol for acute chest pain was modified and monitored for 1 year.

Patients with an ECG that met the criteria for ST-elevation myocardial infarction (STEMI) were immediately transported to a PCI hospital. ECGs that did not meet the STEMI criteria, but showed total ST deviation ≥800 µv were transmitted for online interpretation by the ECG expert. Online supervision was offered as a service if ECGs showed conduction disorders, or had an otherwise ‘suspicious’ pattern according to the ambulance paramedics.

We enrolled 1,076 patients with acute ischaemic chest pain who did not meet the automated STEMI criteria. Their mean age was 63 years; 64% were men. After online consultation, 735 (68%) patients were directly transported to a PCI hospital for further treatment. PCI within 90 min was performed in 115 patients.

**Conclusion:**

During a 1-year evaluation of the modified pre-hospital triage protocol for patients with acute ischaemic chest pain, over 100 acute MI patients with an initially inconclusive ECG received primary PCI within 90 min. Because of these results, we decided to continue the operation of the modified protocol.

## What’s new

- Since the timely identification of MI patients is a universal challenge, we have improved the sensitivity to identify acute MI patients with the modified pre-hospital triage protocol, which combines automated ECG interpretation with on-line expert consultation.
- Over 100 acute MI patients with an initially inconclusive ECG received primary PCI within 90 min.
- It is equally important to filter out normal ECGs. In our study, 64% of the Category 1, 3 and 4 patients that were immediately transported to a PCI centre after on-line supervision by the ECG expert did not undergo revascularisation during hospitalisation. This ‘false positive’ rate is considered acceptable.

## Introduction

In patients presenting with acute chest pain suggestive of ongoing myocardial infarction (MI) early diagnosis and revascularisation treatment leads to favourable clinical outcomes. Patients with ST-elevation myocardial infarction (STEMI) benefit most from percutaneous coronary intervention (PCI) when performed within 2 h after symptom onset [1–3]. In the Netherlands, early mortality was reported to be as low as 1.6% in patients who receive PCI treatment in the first hour after symptom onset, compared with 4.0% in those treated after 5 h [4]. Similarly low mortality has been reported after early PCI in patient with non-ST-elevation acute coronary syndrome (NSTE-ACS) with a so-called ‘high-risk profile’, including patients with a Global Registry of Acute Coronary Events (GRACE) risk score >140 [5, 6]. Thus, minimising total ischaemic time is the key to improve the prognosis of STEMI patients and high-risk NSTE-ACS patients, which is mainly a logistical challenge that starts in the pre-hospital setting.

For decades, the standard 12-lead electrocardiogram (ECG) has been the main diagnostic tool in the assessment of patients with acute chest pain. Worldwide, in the majority of patients presenting with symptoms suggestive of ongoing MI in a pre-hospital setting an ECG will be obtained by the emergency medical service (EMS). Ambulances in the Netherlands are staffed by paramedics and equipped with a patient-monitoring device that is capable not only to derive and register such ECGs, but also to provide an automated analysis and interpretation. Patients with an ECG that is interpreted as ‘evolving MI’ are then directly transported with highest emergency for coronary angiography and revascularisation therapy to the nearest hospital with PCI service. Patients with an inconclusive ECG are transported to non-PCI hospitals for further diagnosis and treatment.

Satisfying results have been reported in relation to the implementation of ECG-based triage protocols [3], also in the Rotterdam-Rijnmond region, the Netherlands, albeit in the thrombolysis era [7]. Still, in pre-hospital settings, it remains challenging to adequately identify those patients who require immediate reperfusion therapy when automated ECG analysis provides inconclusive results. In the past years, we have obtained anecdotic reports that patients with acute ischaemic chest pain who initially were transported to a non-PCI centre in our region needed immediate PCI after all. Review of their medical records showed that the automated ECG interpretation fell short to recognise the ongoing MI, and, consequently, symptom-onset-to-reperfusion times exceeded the guideline-recommended treatment criteria.

Because of these reports, we decided to change the logistic system in our region in December 2013. The automated ECG devices on the ambulances were then supplemented with a modem, which enabled e‑transmission of the ECGs for expert consultation. We modified the diagnostic protocol, utilising this technical option, and we hypothesised that a substantial portion of MI patients would get on the right track to PCI and primary PCI faster. The implementation of the new protocol was monitored during a one-year period, and this paper presents the main findings.

## Methods

### Setting

At the start of this study (in 2013), the Rotterdam-Rijnmond region in the western part of the Netherlands holds a population of 1.1 mio. The region has a total of 10 hospitals, two of which (*Erasmus MC* and *Maasstad Ziekenhuis*) offer a 24/7 primary PCI service for MI patients. The majority of patients with acute ischaemic chest pain have their first medical contact with a paramedic of the ambulance crew—it should be noted that, in the Netherlands a medical doctor is not present on the ambulance. The paramedic performs a brief physical examination and provides an initial diagnosis, which is mainly based on an automated analysis of the ECG. All ambulances in the region are equipped with the Corpuls 3 defibrillator/monitoring system, in which Biosigna HES PRO ECG-interpretation software (algorithm Rev. 2.2) was implemented. Patients with a confirmed evolving MI are then transported to a PCI hospital, whereas the remaining patients are transported to the nearest non-PCI hospital.

### Automated ECG interpretation

**Fig. 1:**
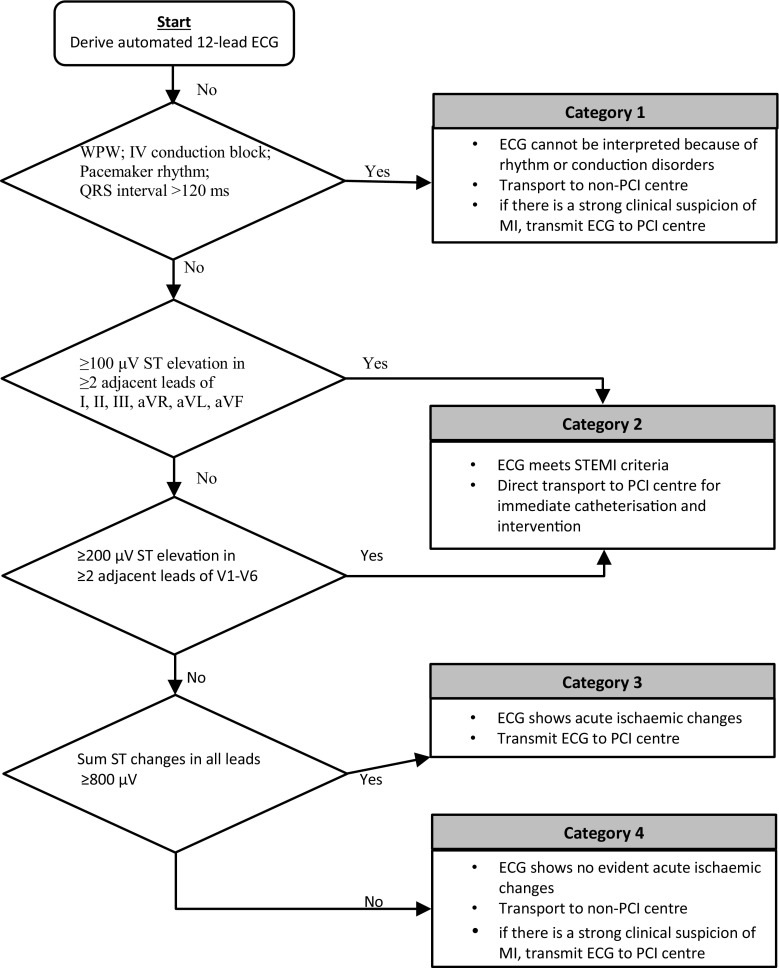
The ECG protocol for pre-hospital myocardial infarction diagnosis. The following categories are distinguished by the automated ECG analysis and interpretation: Category 1: ECG with rhythm or conduction disorders. Normally the patient is transported to a centre without facilities for PCI. If there is a strong clinical suspicion of evolving MI, the ECG will be transmitted for online interpretation by the ECG expert. Category 2: ECG shows ST elevation ≥200 µv in ≥2 adjacent anterior leads, or ≥100 µv in ≥2 non-anterior leads. The ECG meet the criteria for STEMI. Immediate revascularisation is indicated, and patients are directly transported to a PCI hospital. The ambulance staff sends an alert to the PCI hospital, and transmits the ECG for completion of the medical dossier. Category 3: ECGs that do not meet the STEMI criteria, but still show total ST deviation ≥800 µv must now be transmitted for online interpretation by the ECG expert. Category 4: Abnormal ECGs without evident acute ischaemic changes. As in Category 1, the patient is transported to a non-PCI centre. If there is a strong clinical suspicion of evolving MI, the ECG will be transmitted for online interpretation by the ECG expert (*ECG* electrocardiogram, *IV* intraventricular, *MI* myocardial infarction, *PCI* percutaneous coronary intervention, *STEMI* ST-elevation myocardial infarction, *WPW* Wolff-Parkinson-White)

In November 2013, the Corpuls devices on the ambulances in the Rotterdam-Rijnmond region were equipped with modems which enabled transmission of the ECGs for online interpretation by an ECG expert: the on-call cardiologist or cardiology resident in one of the two PCI hospitals (Erasmus MC and Maasstad Ziekenhuis). Since then, the ECG protocol for pre-hospital MI diagnosis in patients with acute ischaemic chest pain (which is a prerequisite) is as follows (Fig. 1).

The existing protocol remained unchanged for patients with an ECG showing ST elevation ≥200 µv in ≥2 adjacent anterior leads, or ≥100 µv in ≥2 non-anterior leads (Category 2). They are directly transported to a PCI hospital, as they meet the STEMI criteria and need immediate revascularisation. The ambulance staff sends an alert to the PCI hospital, and transmits the ECG for completion of the medical record.

With respect to the treatment of other patients, the existing protocol was extended. ECGs that do not meet the STEMI criteria, but still show total ST deviation ≥800 µv (Category 3) must now be transmitted for online interpretation by the ECG expert. In accordance with the advice of the ECG expert, provided by telephone within 5 min, the patient is then transported to the on-call PCI hospital for immediate angiography, possibly followed by revascularisation, or to a non-PCI hospital for further evaluation by a cardiologist. For patients with an ECG that cannot be interpreted by the ECG-interpretation software because of conduction disorders (Category 1), as well as for patients with abnormal ECGs, but not showing evident acute ischaemic changes (Category 4), the ECG expert offers online supervision as a service that is not mandatory.

### Study patients and data collection

We monitored the revised protocol during the 1‑year period from December 2013 to November 2014. Transmitted ECGs and primary data were collected by the ambulance personnel, including age, sex, ECG transmission date and time, and the diagnostic classification that was generated by the Corpuls device. Secondary data were collected by the first author (SA), based on a review of hospital medical records, and included medical history, risk factors, reperfusion time, final discharge diagnosis, and other pertinent clinical outcomes. All data were recorded in a dedicated database.

The patients whose ambulance ECG did not meet the STEMI criteria were the population of main interest (Categories 1, 3, and 4). Still, we also collected information on patients who met the STEMI criteria (Category 2), and whose ECGs were transmitted.

### Study endpoints

The revised protocol was developed to increase the early rule-in of MIs, and to increase the number of patients undergoing ‘primary’ PCI within the recommended 90-minutes window (PCI_90min_), in particular in those patients who initially did not meet the STEMI criteria. PCI_90min_ was therefore defined as the main study endpoint. PCI delay was defined as the time difference between the acquirement of the ECG (time zero) and the wire crossing. The revised protocol also aimed to avoid unnecessary patient burden, invasive diagnostics (coronary catheterisation) and treatments. From this perspective, we considered patients who were immediately transported to a PCI centre but did not undergo PCI during the initial hospitalisation as ‘false positives’. Consequently, PCI_hospitalisation_ was defined as the secondary endpoint. PCI_90min_ is an inappropriate endpoint in this respect, since it will be influenced by logistic delays.

We classified patients according to their final diagnosis as acute MI (STEMI), NSTEMI, unstable angina pectoris (UAP), or ‘other’. The diagnostic and treatment criteria that were used by the treating physicians were based on prevailing European Society of Cardiology guidelines [8–10]. This study was embedded in the clinical practice of the ambulance service and hospitals in the Rotterdam-Rijnmond region, and we accepted the final diagnosis made by the treating cardiologist. We derived this information from the hospital discharge letter, and we did not install an adjudication board to evaluate diagnoses and treatment decisions.

### Statistical analysis

We compared baseline characteristics between the four diagnostic categories. Continuous variables are presented as mean value ± standard deviation (SD) and categorical variables are presented as numbers and percentages.

PCI_90min_ and PCI_hospitalisation_ are reported in relation to the diagnostic category. We conducted logistic regression analyses to examine the relation between diagnostic category and patient characteristics as predictor variables, and PCI_hospitalisation_ as outcome (Category 2 patients are excluded from this analysis). Results are presented as unadjusted and adjusted odds ratios (OR) with 95% confidence intervals (CI). These analyses might be useful to identify patient categories in which the diagnostic system was apparently and definitely—as by judgment of the treating physician—unsuccessful.

Data were analysed with SPSS software (SPSS 23.0 IBM corp., Armonk, NY, USA). Statistical tests were two-tailed and *p*-values <0.05 were considered statistically significant.

### Ethics

This is an observational study. For the purpose of this study, patients were not subject to acts, or imposed to any mode of behaviour, other than standard treatment. For that reason, according to Dutch law, written informed consent for a patient to be enrolled in this study was not necessary. This study was conducted in accordance with the privacy policy of the Erasmus MC, and according to the Erasmus MC regulations for the appropriate use of data in patient-oriented research.

## Results

### Patient characteristics

The study cohort comprised 1,421 patients with a mean age of 62 ± 17 years, and 67% were men (Table 1). A total of 345 patients met the STEMI criteria (Category 2 patients). As compared with the other categories (1, 3, 4), patients from Category 2 were younger (mean age 57 versus 61–68 years), whereas the percentage of men was higher (76 versus 61–72%). Furthermore, these patients had a somewhat more favourable cardiovascular disease risk profile, as fewer patients had hypertension (37 versus 58–64%), hypercholesterolaemia (29 versus 43–47%), diabetes mellitus (11 versus 22–25%) and a history of coronary artery disease (CAD) (17 versus 32–43%).

**Table 1:** Baseline characteristics according to the automated ECG-based initial diagnosis

		Automated ECG-based initial diagnosis				
	All patients	Category 1	Category 2	Category 3	Category 4	*P*-value
		ECG cannot be interpreted because of rhythm disturbances	ECG meets STEMI criteria	ECG shows acute ischaemic changes	ECG shows no evident acute ischaemic changes	
No. of patients	1,421	228	345	526	322	
*Demographic characteristics*						
Age, years	62 ± 17	68 ± 14	57 ± 17	62 ± 18	61 ± 15	<0.001
Men	67	72	76	61	64	<0.001
*Cardiovascular risk factors* ^*a*^						
Hypertension	53	64	37	58	60	<0.001
Hypercholesterolaemia	41	47	28	43	48	<0.001
Diabetes mellitus	20	25	11	23	22	<0.001
Current smoker	34	27	47	33	40	<0.001
Positive family history	36	28	42	33	40	0.021
*Cardiovascular history* ^*a*^						
CAD	31	43	17	32	38	<0.001
MI	22	30	14	19	31	<0.001
PCI	20	22	13	17	29	<0.001
CABG	7	11	1	9	8	<0.001
AF	10	17	2	12	9	<0.001

Data represent mean ± standard deviation (SD) values or percentages

^a^Data on cardiovascular risk factors and cardiovascular history were only available for the 1,022 (72%) patients who were directly transported to a PCI centre. Data on smoking was complete in 89% and data on family history of coronary disease in 87% of patients

Category 1: ECG with rhythm or conduction disorders. Category 2: ECG that shows ST elevation ≥200 µv in ≥2 adjacent anterior leads, or ≥100 µv in ≥2 non-anterior leads. Category 3: ECG that show total ST deviation ≥800 µv. Category 4: abnormal ECG, without evident acute ischaemic changes

*AF* atrial fibrillation, *CABG* coronary artery bypass grafting, *CAD* coronary artery disease, *ECG* electrocardiogram, *MI* myocardial infarction, *PCI* percutaneous coronary intervention

### Initial diagnostic category and treatment decisions

As Table 2 demonstrates, a total of 287 (83%) of Category 2 patients were directly transported to a PCI hospital. The reasons why the remaining 17% stayed home or were transported to a non-PCI hospital were not recorded. The final diagnosis acute MI with STEMI was made in 222 (77%). Twelve patients (4%) had NSTEMI (the cardiac enzymes were positive without further increased STEMI abnormalities on the ECG) or UAP. Other diagnoses included pre-existing STEMI abnormalities on the ECG, pericarditis, costo-myalgia or cardiomyopathy. In STEMI patients, PCI_hospitalisation_ was performed in 211 (95%) cases, whereas 73% had PCI_90min_. A total of 8 (67%) NSTEMI/UAP patients had PCI_hospitalisation_, and 25% had PCI_90min_.

**Table 2:** Final diagnosis and treatment according to the automated ECG-based initial diagnosis

	All patients	Category 1	Category 2	Category 3	Category 4	*P*-value
		ECG cannot be interpreted because of rhythm disturbances	ECG meets STEMI criteria	ECG shows acute ischaemic changes	ECG shows no evident acute ischaemic changes	
ECG transmitted to expert	1,421	228	345	526	322	–
Direct transport to PCI centre after expert supervision	1,022 (72)	182 (80)	287 (83)	333 (63)	220 (68)	<0.001
*Final diagnosis*						
Acute MI	431 (42)	76 (42)	222 (77)	85 (26)	48 (22)	<0.001
NSTEMI/UAP	144 (14)	31 (17)	12 (4)	69 (21)	32 (15)	
Other	447 (44)	75 (41)	53 (19)	179 (54)	140 (64)	
*PCI performed in Acute MI* ^*a*^						
<90 min	263/385 (68)	42/68 (62)	148/202 (73)	49/70 (70)	24/45 (53)	0.007
<120 min	300/385 (78)	52/68 (76)	165/202 (82)	53/70 (76)	30/45 (67)	0.013
During hospitalisation	400 (93)	71 (93)	211 (95)	72 (85)	46 (96)	0.073
*PCI performed in NSTEMI/UAP*						
<90 min	14/86 (16)	1/17 (6)	2/8 (25)	8/44 (18)	3/17 (18)	0.827
<120 min	16/86 (19)	2/17 (12)	2/8 (25)	9/44 (20)	3/17 (18)	0.775
During hospitalisation	86 (60)	17 (55)	8 (67)	44 (64)	17 (53)	0.632

Data represent numbers (percentages)

^a^PCI was performed in 385 acute MI patients. The other 46 (11%) of 431 acute MI patients had no indication for PCI due to medical conditions or other circumstances such as age (>87 years), multivessel disease, preferred for CABG treatment based on occlusion of multiple cardiac blood vessels or other medical history

Category 1: ECG with rhythm or conduction disorders. Category 2: ECG that shows ST elevation ≥200 µv in ≥2 adjacent anterior leads, or ≥100 µv in ≥2 non-anterior leads. Category 3: ECG that show total ST deviation ≥800 µv. Category 4: abnormal ECG, without evident acute ischaemic changes

*CABG* coronary artery bypass grafting, *ECG* electrocardiogram, *NSTEMI* non-ST-elevation myocardial infarction, *PCI* percutaneous coronary intervention, *STEMI* ST-elevation myocardial infarction, *UAP* unstable angina pectoris

**Fig. 2:**
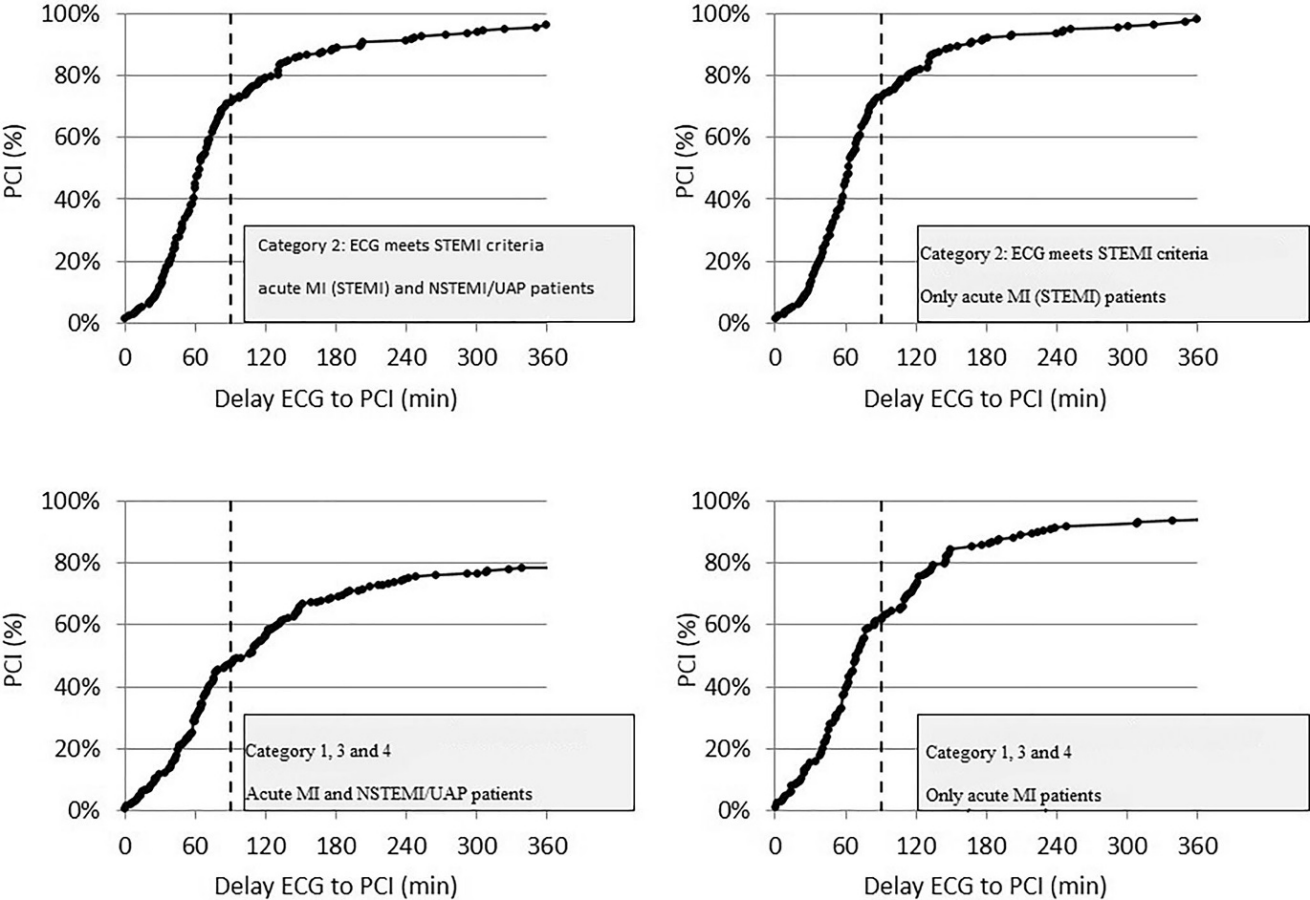
Time delay between ECG transmission and percutaneous coronary intervention treatment. The results are presented as PCI (percentages) time delay (per minute) between the ECG transmission time and PCI treatment time for patients in Category 2 and the Categories 1, 3 and 4 combined. Category 1: ECG with rhythm or conduction disorders. Category 2: ECG that shows ST elevation ≥200 µv in ≥2 adjacent anterior leads, or ≥100 µv in ≥2 non-anterior leads. Category 3: ECG that shows total ST deviation ≥800 µv. Category 4: abnormal ECG, without evident acute ischaemic changes (*ECG* electrocardiogram, *MI* myocardial infarction, *PCI* percutaneous coronary intervention, *NSTEMI* non-ST-elevation myocardial infarction, *STEMI* ST-elevation myocardial infarction, *UAP* unstable angina pectoris)

After online consultation with the ECG expert, 735 (68%) Category 1, 3 and 4 patients were directly transported to a PCI hospital for catheterisation and further treatment. A total of 209 (28%) were diagnosed with acute MI and 132 (18%) patients were diagnosed NSTEMI (positive cardiac enzyme) or UAP. An indication for PCI was present in 189 acute MI patients. The remaining patients had contraindication for PCI because of advanced age, multiple physical comorbidities or had an indication for coronary artery bypass grafting (CABG) treatment. PCI_90min_ was performed in 63% and 15% of acute MI and NSTEMI/UAP patients, respectively. The percentage of patients with a final diagnosis of acute MI ranged from 42% in Category 1 to 22% in Category 4, whereas, in these acute MI patients, PCI_90min_ ranged from 53% (Category 4) to 70% (Category 3). Fig. 2 shows details of time delays between ECG transmission and PCI treatment. Apparently, delays were longer for patients with an initial ECG that did not meet the STEMI criteria.

### Determinants of PCI_hospitalisation_ treatment

Table 3 shows determinants of PCI_hospitalisation_. Patients presenting with an initial ECG that shows rhythm or conduction disturbances, which for that reason could not be analysed by the ECG-interpretation software, had considerably higher odds to receive PCI_hospitalisation_ than those with abnormal, but interpretable ECGs (49 versus 30%, OR_adjusted_ 2.7). Interestingly, patients with a history of atrial fibrillation had apparently lower odds for PCI_hospitalisation_ than patients with normal rhythm (20 versus 41%, OR_adjusted_ 0.25). Women had lower odds than men (31 versus 42%, OR_adjusted_ 0.56), whereas elderly patients had higher odds (OR_adjusted_ 1.04 per year). Also smoking status and a positive family history of CAD appeared to be related to PCI_hospitalisation_ treatment.

**Table 3:** Relation between patient characteristics and in-hospital percutaneous coronary intervention

		In-hospital PCI, %	Crude odds ratio (95% CI)	Adjusted odds ratio (95% CI)^a^	*P*-value
Automated ECG-based initial diagnosis	Category 1^b^	49	2.3 (1.5, 3.5)	2.7 (1.6, 4.5)	<0.001
	Category 3^b^	37	1.4 (0.96, 2.0)	1.5 (0.98, 2.3)	0.077
	Category 4^b^	30	1	1	
*Demographic characteristics*					
Gender	Women	31	0.62 (0.45, 0.86)	0.56 (0.37, 0.84)	0.006
	Men	42	1	1	
Age, years			1.01 (1.00, 1.02)	1.04 (1.02, 1.05)	<0.001
*Cardiovascular risk factors*					
Hypertension	Yes	35	0.75 (0.55, 1.0)	0.80 (0.50, 1.3)	0.35
	No	42	1	1	
Hypercholesterolaemia	Yes	35	0.82 (0.61, 1.1)	1.0 (0.61, 1.8)	0.89
	No	40	1	1	
Diabetes mellitus	Yes	32	0.69 (0.48, 1.0)	0.68 (0.43, 1.1)	0.10
	No	40	1	1	
Current smoker	Yes	48	1.6 (1.1, 2.2)	1.5 (1.0, 2.2)	0.044
	No	37	1	1	
Positive family history	Yes	49	1.7 (1.2, 2.4)	2.3 (1.5, 3.3)	<0.001
	No	36	1	1	
*Cardiovascular history*					
CAD	Yes	33	0.69 (0.50, 0.95)	0.69 (0.36, 1.3)	0.27
	No	41	1	1	
MI	Yes	34	0.79 (0.56, 1.1)	0.90 (0.47, 1.7)	0.75
	No	40	1	1	
PCI	Yes	35	0.84 (0.59, 1.2)	0.88 (0.48, 1.6)	0.67
	No	39	1	1	
CABG	Yes	25	0.50 (0.28, 0.90)	0.49 (0.22, 1.1)	0.080
	No	39	1	1	
AF	Yes	20	0.37 (0.22, 0.64)	0.25 (0.12, 0.53)	<0.001
	No	41	1	1	

^a^Based on a logistic regression model, including all factors in the Table

^b^Category 1: ECG with rhythm or conduction disorders. Category 3: ECG that show total ST deviation ≥800 µv. Category 4: abnormal ECG, without evident acute ischaemic changes

*AF* atrial fibrillation, *CABG* coronary artery bypass grafting, *CAD* coronary artery disease, *ECG* electrocardiogram, *MI* myocardial infarction, *PCI* percutaneous coronary intervention

## Discussion

During a 1-year evaluation of the modified pre-hospital triage protocol for patients with acute ischaemic chest pain in the Rotterdam-Rijnmond region, 115 acute MI patients with an initially inconclusive ECG received primary PCI within 90 min, whereas another 20 received PCI within 90–120 min. Because of these results, we have decided to continue the operation of the modified protocol.

We initiated our project because we obtained anecdotical reports of patients with acute ischaemic chest pain in our region with an initially inconclusive ECG, who were transported to a non-PCI centre, and who ultimately underwent immediate PCI for acute MI. We intentionally designed an implementation study, and not a randomised trial, neither an observational before-after study. Accordingly, we cannot conclude with entire certainty that the observed early treatment was the direct consequence of a change in patient flow that was induced by the new triage protocol. Still, however, it must be appreciated that the original protocol recommended that these patients be transferred to a regional non-PCI hospital for further evaluation, whereas Miedema et al. observed that inter-hospital transfer was the most frequent cause of treatment delay in STEMI patients [11]. Wang et al. demonstrated in the Acute Coronary Treatment and Intervention Outcomes Network (ACTION) registry that door-in-door-out times from non-PCI to PCI hospitals might be as long as 68 min in 50% of patients [12]. Prior studies showed that <10% of STEMI patients with inter-hospital transfer were treated within 90 min and only 15–36% within 120 min [13]. These data support the benefits of the modified prehospital triage protocol in our region.

Several studies support the use of pre-hospital ECGs to reduce ischaemic times in patients presenting with STEMI or NSTE-ACS [14–16]. Health care systems that involve trained paramedics for ECG interpretation [17, 18], as well as systems that implemented automated ECG interpretation [19, 20] had satisfactory diagnostic performance and ditto beneficial results. Nevertheless, it has been demonstrated that a cardiologist or medical doctor overview and confirmation improves diagnostic accuracy [21], while treatment delays are not increased [22]. In particular, ECG artefacts will then be avoided [23]. Our observation of an improved sensitivity to diagnose acute MI through the combination of automated ECG interpretation and expert consultation is in agreement with these studies.

It is equally important to filter out normal ECGs to avoid unnecessary treatments and overcrowding at the PCI hospital [24, 25]. In our study, 64% of the Category 1, 3 and 4 patients who were immediately transported to a PCI centre after online supervision by the ECG expert did not undergo revascularisation during hospitalisation. Apparently, CAD requiring immediate treatment, and thus evolving MI, was excluded by the treating physician. In view of the observed benefits, we consider the 36/64 ratio acceptable, although there is room for improvement. Adding diagnostic and risk stratification tools could be helpful in this respect. We found that PCI_hospitalisation_ was less likely in women, in younger patients, in non-smokers and in those without a family history of CAD. Still, differences were not considerable enough to justify a stratified approach according to these characteristics. The application of established risk stratifications scores in the pre-hospital setting, such as the thrombolysis in myocardial infarction (TIMI) risk score, GRACE risk score, or the history, ECG, age, risk factor and troponin (HEART) score might be beneficial to improve the diagnostic system [26–28]. Finally, research is warranted to evaluate the diagnostic performance of the combination of automated ECG interpretation and out-of-hospital point-of-care troponin tests, which recently have become available [29, 30].

### Limitations

As by design, our study has several limitations that need to be mentioned. First, the telephone conversation between the ambulance paramedic and the on-call ECG expert was neither protocolised nor reported. As a result, we could not evaluate the factors that actually affected the reason for acceptance or refusal for immediate transportation to the PCI centre. This is particular relevant for patients living in the zip code area of the PCI-capable hospital. Most likely, the threshold to undergo early CAG in these patients is lower than for their counterparts living at further distance. Second, it is possible that the phone call, in which the clinical condition was discussed, led to the admission, and not the transmitted and reviewed ECG per se. Unfortunately, however, we are not able to disentangle the influence of both phenomena. Third, we did not follow up the patients who stayed at home, or who were transported to a non-PCI centre. We appreciate that patients who ultimately had MI might still have been missed in the prehospital phase, but, consequently, we cannot quantify their number. This is particularly the case for patients who were labelled by the ECG-interpretation software as Category 1 or 4. Fourth, we did not measure clinical outcomes. Thus—apart from the fact that our study is not a randomised trial—we are not able to demonstrate the benefit of the revised diagnostic system in terms of patient outcomes.

### Conclusion

In conclusion, by applying the modified pre-hospital triage protocol for patients with acute ischaemic chest pain in the Rotterdam-Rijnmond region, during a 1-year period, over 100 acute MI patients with an initially inconclusive ECG received primary PCI within 90 min. The ‘false positive’ rate of 64% is considered acceptable. Still, further research is warranted to improve the specificity of the triage protocol, so that unnecessary burden to the patient and the system will be avoided.

## References

[1] Terkelsen CJ, Sorensen JT, Maeng M (2010). System delay and mortality among patients with STEMI treated with primary percutaneous coronary intervention. JAMA.

[2] Rathore SS, Curtis JP, Chen J (2009). Association of door-to-balloon time and mortality in patients admitted to hospital with ST elevation myocardial infarction: national cohort study. BMJ.

[3] Carstensen S, Nelson GC, Hansen PS (2007). Field triage to primary angioplasty combined with emergency department bypass reduces treatment delays and is associated with improved outcome. Eur Heart J.

[4] Fokkema ML, Wieringa WG, van der Horst IC (2011). Quantitative analysis of the impact of total ischemic time on myocardial perfusion and clinical outcome in patients with ST-elevation myocardial infarction. Am J Cardiol.

[5] Jobs A, Mehta SR, Montalescot G (2017). Optimal timing of an invasive strategy in patients with non-ST-elevation acute coronary syndrome: a meta-analysis of randomised trials. Lancet.

[6] Milosevic A, Vasiljevic-Pokrajcic Z, Milasinovic D (2016). Immediate versus delayed invasive intervention for non-STEMI patients: the RIDDLE-NSTEMI study. JACC Cardiovasc Interv.

[7] Boersma E, Maas AC, Hartman JA, Ilmer B (2001). [Twelve year triage and thrombolysis treatment prior to hospitalization for myocardial infarction patients in the Rotterdam area of the Netherlands: outstanding short-term and long-term results] 12 jaar triage en trombolytische behandeling voor ziekenhuisopname bij hartinfarctpatienten in de regio Rotterdam: uitstekende korte- en langetermijnresultaten. Ned Tijdschr Geneeskd.

[8] Ibanez B, James S, Agewall S, et al. 2017 ESC Guidelines for the management of acute myocardial infarction in patients presenting with ST-segment elevation: The Task Force for the management of acute myocardial infarction in patients presenting with ST-segment elevation of the European Society of Cardiology (ESC). Eur Heart J. 2018;39(2):119–177. 10.1093/eurheartj/ehx393 28886621

[9] Roffi M, Patrono C, Collet JP (2016). 2015 ESC Guidelines for the management of acute coronary syndromes in patients presenting without persistent ST-segment elevation: Task Force for the Management of Acute Coronary Syndromes in Patients Presenting without Persistent ST-Segment Elevation of the European Society of Cardiology (ESC). Eur Heart J.

[10] Steg PG, James SK, Atar D (2012). ESC Guidelines for the management of acute myocardial infarction in patients presenting with ST-segment elevation. Eur Heart J.

[11] Miedema MD, Newell MC, Duval S (2011). Causes of delay and associated mortality in patients transferred with ST-segment-elevation myocardial infarction. Circulation.

[12] Wang TY, Nallamothu BK, Krumholz HM (2011). Association of door-in to door-out time with reperfusion delays and outcomes among patients transferred for primary percutaneous coronary intervention. JAMA.

[13] Chakrabarti A, Krumholz HM, Wang Y, National Cardiovascular Data Registry (2008). Time-to-reperfusion in patients undergoing interhospital transfer for primary percutaneous coronary intervention in the U.S: an analysis of 2005 and 2006 data from the National Cardiovascular Data Registry. J Am Coll Cardiol.

[14] Quinn T, Johnsen S, Gale CP (2014). Effects of prehospital 12-lead ECG on processes of care and mortality in acute coronary syndrome: a linked cohort study from the Myocardial Ischaemia National Audit Project. Heart.

[15] Cheskes S, Turner L, Foggett R (2011). Paramedic contact to balloon in less than 90 minutes: a successful strategy for st-segment elevation myocardial infarction bypass to primary percutaneous coronary intervention in a canadian emergency medical system. Prehosp Emerg Care.

[16] Daudelin DH, Sayah AJ, Kwong M (2010). Improving use of prehospital 12-lead ECG for early identification and treatment of acute coronary syndrome and ST-elevation myocardial infarction. Circ Cardiovasc Qual Outcomes.

[17] O’Donnell D, Mancera M, Savory E (2015). The availability of prior ECGs improves paramedic accuracy in recognizing ST-segment elevation myocardial infarction. J Electrocardiol.

[18] Lee CH, Van Gelder CM, Cone DC (2010). Early cardiac catheterization laboratory activation by paramedics for patients with ST-segment elevation myocardial infarction on prehospital 12-lead electrocardiograms. Prehosp Emerg Care.

[19] Potter BJ, Matteau A, Mansour S (2017). Sustained performance of a “physicianless” system of automated prehospital STEMI diagnosis and catheterization laboratory activation. Can J Cardiol.

[20] Bradley EH, Herrin J, Wang Y (2006). Strategies for reducing the door-to-balloon time in acute myocardial infarction. N Engl J Med.

[21] Huitema AA, Zhu T, Alemayehu M, Lavi S (2014). Diagnostic accuracy of ST-segment elevation myocardial infarction by various healthcare providers. Int J Cardiol.

[22] Mawri S, Michaels A, Gibbs J (2016). The comparison of physician to computer interpreted electrocardiograms on ST-elevation myocardial infarction door-to-balloon times. Crit Pathw Cardiol.

[23] Bosson N, Sanko S, Stickney RE (2017). Causes of prehospital misinterpretations of ST elevation myocardial infarction. Prehosp Emerg Care.

[24] Dieker HJ, Liem SS, El Aidi H (2010). Pre-hospital triage for primary angioplasty: direct referral to the intervention center versus interhospital transport. JACC Cardiovasc Interv.

[25] Larson DM, Menssen KM, Sharkey SW (2007). “False-positive” cardiac catheterization laboratory activation among patients with suspected ST-segment elevation myocardial infarction. JAMA.

[26] Van Den Berg P, Body R (2017). The HEART score for early rule out of acute coronary syndromes in the emergency department: a systematic review and meta-analysis. Eur Heart J Acute Cardiovasc Care.

[27] Antman EM, Cohen M, Bernink PJ (2000). The TIMI risk score for unstable angina/non-ST elevation MI: a method for prognostication and therapeutic decision making. JAMA.

[28] Granger CB, Goldberg RJ, Dabbous O (2003). Predictors of hospital mortality in the global registry of acute coronary events. Arch Intern Med.

[29] Kip MMA, Koffijberg H, Moesker MJ (2017). The cost-utility of point-of-care troponin testing to diagnose acute coronary syndrome in primary care. BMC Cardiovasc Disord.

[30] Van Hise CB, Greenslade JH, Parsonage W (2018). External validation of heart-type fatty acid binding protein, high-sensitivity cardiac troponin, and electrocardiography as rule-out for acute myocardial infarction. Clin Biochem.

